# Narratives of Obstetric Violence: Conceptions of Power, Bodily Integrity, and Childbirth in Greek Women's Stories

**DOI:** 10.1177/10778012251369025

**Published:** 2025-08-14

**Authors:** Faidra Gatsarouli, Linda Berg

**Affiliations:** 8075 Umea University, Umeå, Sweden

**Keywords:** obstetric violence, feminist phenomenology, Greece, birthing body, qualitative research

## Abstract

This qualitative study examines women's narratives of obstetric violence in Greece, highlighting how power structures, medicalized childbirth, and cultural norms shape both the experience and normalization of such violence. By analyzing 63 participants’ responses to an online survey with seven open-ended questions, the study applies thematic analysis and feminist phenomenology to explore how obstetric violence affects women's bodies, relations and self-perception. The findings reveal not only its pervasiveness and systematic invisibility, often justified as routine care, but also its entrenchment within sociocultural and institutional frameworks. However, it also identifies pathways for resistance and healing through community, advocacy, and collective action.

## Introduction

Giving birth is a personal embodied experience that every birthing subject should be able to own and (re)claim. Recognizing abuse in obstetric care, particularly as gendered violence, has historically been a challenging and ongoing fight for feminist activists and movements (Cohen Shabot, 2016; Savage & Castro, 2017). However, formal legal recognition and status have recently been granted to this issue. The World Health Organization (WHO, 2014) defines obstetric violence as:

the disrespectful and abusive treatment during childbirth in facilities that includes outright physical abuse, profound humiliation, and verbal abuse, coercive or unconsented medical procedures (including sterilization), lack of confidentiality, failure to get fully informed consent, refusal to give pain medication, gross violations of privacy, refusal of admission to health facilities, neglecting women during childbirth to suffer life-threatening, avoidable complications, and detention of women and their newborns in facilities after childbirth due to an inability to pay. (p1)

Feminist scholars (Cohen Shabot, 2016; Rich, 1995; Van Der Waal et al., 2021; Young, 1984) connect obstetric violence with the appropriation of the female body; it involves the violation of a woman's bodily autonomy and her right to make decisions about her own well-being during pregnancy, childbirth, and postpartum (Cohen Shabot, 2016, 2021; Morgan & Björkert, 2006; Savage & Castro, 2017). When subjected to obstetric violence, a woman's agency is often disregarded as her body becomes an object upon which medical professionals act without considering her desires and needs. In this way, the phenomenon perpetuates a patriarchal ideology that seeks to appropriate and govern female bodies. “Babies are described as being ‘delivered’ by the medical staff, silencing not only the woman's body but also her voice as an expectant mother, a laboring woman, and a patient” (Cohen Shabot & Korem, 2018, p. 391).

Regarding Greece, women commonly gave birth at home, allowing them to have ownership and control over the event. However, as the state underwent economic and societal changes in the 1970s, childbirth became medicalized. This meant that responsibility and control shifted to primarily male physicians, while midwives took on a supportive role (Lefkarites, 1992). As the childbirth setting in Greece has shifted to a medicalized and interventional one (Kontopanos et al., 2023; Papoutsis & Chatzipanagiotidou, 2023), more research on obstetric violence needs to be done.

This study focuses on women's narratives of obstetric violence in Greece, unfolding notions of power, female bodies and childbirth as a biomedicalized process. More specifically, *the aim is to explore how experiences of obstetric violence are intertwined in conceptions of gender, bodily integrity and reproduction in a Greek context*. Following a qualitative methodology and a feminist phenomenological approach, it focuses on two research questions: how do women as embodied subjects experience obstetric violence and how its aftermath shapes their relationship with their bodies, emotional resilience, and sense of justice within social connections?

An important ambition with this research is to contribute to the broader discussion on how systemic power dynamics shape embodied experiences of obstetric violence. Here, one objective was to hold a position of critique (similar to Sara Ahmed's feminist killjoys), questioning whose knowledge is deemed legitimate inside the maternity wards by amplifying a selection of Greek women's voices and experiences (cf. Ahmed, 2010).

## Recapturing Obstetric Violence

Since the early 2000s, the term obstetric violence has taken a wide recognition academically and legally, indicating the extension, predominance, and significance of the phenomenon (Castro & Frías, 2020). Chadwick (2021) argues that defining obstetric violence remains a contested issue. Rooted in racism and sexism, it was long hidden under colonial structures (Davis, 2019; Van Der Waal et al., 2021). Definitions may often highlight how birthing practices are overly technocratic while neglecting certain forms of harm, such as the underuse of medical interventions when needed (Chadwick, 2021).

Lappeman and Swartz (2019) reflect critically on the term, opening the discussion on reframing violence in obstetric care. Taking into consideration the sociopolitical factor, they question the characterization of inadequate care as violent and the potential compliance with Western definitions of appropriate care. While forms of direct and tangible acts of violence should be named as such, forms of “gentle violence” (Chadwick, 2018), such as the discouragement of the presence of birthing partners, may mislead and disempower people and systems to change. Lappeman and Swartz (2021) stand skeptical toward a broadened definition of violence, since this could cause confusion regarding intentionality, influencing how healthcare situations are interpreted and understood. Besides some critical reflections on the phenomenon, the terminology has traveled across transnational boundaries, with activists and scholars from a range of settings adopting the framework (Allers, 2020; Amroussia et al., 2017; Chadwick, 2016; Chattopadhyay et al., 2018; Diaz-Tello, 2016; Dutton & Knight, 2020; Eimai maia, 2018; Garcia, 2020; Keedle et al., 2024; Lappeman & Swartz, 2019; Mihret, 2019; Pickles, 2015; Ravaldi et al., 2018; Sadler et al., 2016; Shrivastava & Sivakami, 2020; Vaitsopoulou, 2022, November 25).

Davis (2019, p. 561) argues that obstetric violence is an “institutionally and state-sanctioned practice,” highlighting the intersectional character of the violence in birthing as a social, ideological, and political phenomenon. This perspective can be linked to arguments regarding technocratic obstetric training (Davis-Floyd, 1987; Van Der Waal et al., 2021), where hospitalized birth functions as a rite of passage for both gestational subjects and medical trainees. Through ritualized overuse of technological interventions, both groups reinforce a strong narrative of the female body's powerlessness during labor, stripping it of its agency and shifting the control to medical professionals. Van Der Waal et al. (2022) suggest that obstetric training goes beyond an initiation to the technomedical model: it enforces a power dynamic where the medical staff dominate and control the experience of childbirth through appropriating the pregnant body. This means the birthing body is approached as inferior within the medical context, as an object lacking knowledge, and thus expected to be docile and silent under the medical authority.

## Birthing, Female Bodies, and Institutionalized Objectification

It is important to acknowledge that obstetric violence is, first and foremost, deeply personal, shaping self-defined women's experiences, perceptions of their bodies, and trust in healthcare. As discussed above, several studies have talked about obstetric violence from legal, medical, or policy perspectives, but dismissed the voices of those who experienced it. In what we see as a larger narrative by women, the struggle to be heard and the feeling of being voiceless and powerless against the imposition of medical rules and norms are some of the recurring themes (Oliveira & Penna, 2017). In a broader sense, it is also important to recall here that childbirth is a core issue for the state and, as such, a site of wide-ranging power struggle of bodies (e.g., Alm & Berg, 2023).

A common finding (Alnabilsy & Sharon, 2023; Oliveira & Penna, 2017) is the opposition between the presumed autonomy of birthing women as is reported by the medical staff, and the painful and vulnerable position that women are immersed in during labor. Feeling exposed and vulnerable, many women experience a deep sense of isolation during childbirth, unable to express their bodily awareness or fulfill their needs. In a hospitalized context full of restrictive expectations, misjudgments, inadequate explanations, and a lack of further silence their pain, many birthing women feel helpless and objectified, passive subjects rather than active participants in their own labor (Alnabilsy & Sharon, 2023).

When entering the hospital, birthing women enter into “a state of institutionalized objectification” (Ladeira & Borges, 2022, p. 8), which is accompanied by a feeling of not belonging to their own bodies, being blamed and held accountable for their loud voices and needs, and having their physical and emotional subjectivities being disregarded. The privilege of knowledge seems to negatively impact the doctor–women relationship, as it is often used as a tool for demonstrating power and enforcing control rather than for support and empowerment. Even when women come with their well-researched birthing plans, their wishes are often dismissed (Ladeira & Borges, 2022).

Although there has been some improvement in hospitalized intrapartum care, the COVID-19 pandemic led to a significant setback. Besides the increased number of unnecessary and unwanted medical interventions, the very tight and restrictive hospital protocols led to a further dehumanization of childbirth through the prohibition of company during birth, the instant separation from the baby, and the discouragement of breastfeeding (Sadler et al., 2020).

The aftermath of obstetric violence often involves physical and psychological trauma. Although the rates for postpartum depression are already high, having a violent experience during labor, lack of support and an NICU newborn can skyrocket these statistics (Çetin et al., 2023; De Souza et al., 2017; Martinez-Vázquez et al., 2022). Many studies (Perrotte et al., 2020) have underlined a loss of trust in the healthcare system, with women having doubts or denial about future childbirth, feelings of helplessness and lack of support, and a sense of detachment from their own bodies and babies. Additionally, many women struggle with guilt over their traumatic experiences while describing the event as rape (Annborn & Finnbogadóttir, 2021).

## Giving Birth in Greece

Giving voice to postbirthing people in Greece who have experienced obstetric violence goes beyond understanding the phenomenon itself. The unique historical interplay of socioeconomic factors that shifted childbirth into a medical process and the longstanding cultural tradition of informal payments in the medical system make this group worth researched. The experiences of Greek women reflect how systemic and cultural factors shape and perpetuate obstetric violence, making it not just a personal experience but a structural issue rooted in broader societal norms and power dynamics.

In contrast to many other countries in Europe, childbirth has been established later in Greece as something that takes place in hospitals. In Sweden, for example, a gradual change began in the early 1900s (J Wisselgren, 2005). The transition from a private home birth to a medically facilitated/hospitalized labor in Greece came along with a broader change in the Greek economy during the 1970s, from farming to an industrialized economy, as well as a shift in the traditional family structure from a strictly private to a more public space (Lefkarites, 1992).

As Lefkarites (1992) describes, home labor used to be a common practice for women in Greece, which allowed them to have ownership and more control over the event. Once birth began being medicalized, responsibility and control were transferred to a male birth physician, while the role of the midwife was shifted from providing the primary care during delivery to assisting the male doctor. This shift of birth location triggered the change in who has control and power over the birthing event and bodies. Once childbirth was transferred to a medical unit, the laboring women had to rely on medical interventions and male doctors and became dependent on them. As a result, women relinquished control over their actions and subsequently became disengaged from actively participating in the birthing process (Lefkarites, 1992).

Georges (1996, p. 160) describes pregnancy and birth in Greece as a “modern medical hegemony.” Knowledge of childbirth was appropriated by science, giving legitimacy only to the bodies that personalized it. As one doctor described it, “the obstetrician's role was to explain to the woman what it means to be pregnant, what's happening inside of her” (Georges, 1996, p. 161). Moreover, Georges (1996) connects the sometimes excessive use of technomedical procedures with the informal payment culture that exists in Greece. This large, informal economy in public maternity hospitals (Kaitelidou et al., 2013) reinforces the authority of the medical occupation; an authority that is exerted even over the state's control mechanism.

Regarding obstetric violence in Greece, research on the phenomenon has been scarce and has not adequately utilized qualitative research methods. A recent quantitative study (Antoniou, 2021) found that 36.9% of the participants had faced obstetric violence, leading to a statement that obstetric violence exists in Greece and needs to be more researched. Data from Athanasios Kontopanos et al. (2023) showed that in 2019–2020, 55.79% of births were delivered via cesarean section and Papoutsis and Chatzipanagiotidou (2023), in a 20-year-long research, found that 42.8% of births involved cesareans, although the majority of labors (78.8%) were low risk. Half of the participants reported restrictions on free movement, whereas the lithotomy position was imposed on 81.4% of the women. Roughly two out of every three women indicated undergoing a vaginal examination at least once per hour, and around 30% experienced more than five such examinations during labor. The results of these two studies illustrate an image of a country in which more than half of the deliveries are carried out via a cesarean section, and a high rate of medical interventions is imposed on nearly eight out of 10 labors.

## The Relational Subjectivity of Violence

The experience of obstetric violence cannot be examined in isolation from the broader social and philosophical frameworks that shape women's subjectivity. For the purpose of this study, feminist phenomenology was chosen as an appropriate theoretical framework, with an emphasis on subjectivity as dynamic and constantly constructed in time and space (e.g., Bartky, 1990; De Beauvoir, 1949; Young, 1980). One's construction of personhood cannot escape the—timely changing and space emerging—social construction(s) that this very same world has upon their bodies as social identities. Being a body implies being connected to a particular world or environment, meaning that one's body is influenced and shaped by the spatial context in which it exists (Merleau-Ponty, 1962). The body is not just physically, materialistically located in space but is inseparable from it, as the “body is not primarily in space: it is of it” (p. 171). “From this perspective, subjectivity is inherently intersubjective. There are neither subjects nor objects which have substance or meaning outside of the field of relations” (Kaku, 2024, p. 7).

When it comes to female subjectivity, patriarchy condemns women to a sense of a Cartesian self, which is valued and tied upon by its materialistic substance. Their existence is tied to “nature, immanence, and the requirements of the species at the expense of [their] own individuality” (Young, 1980, p. 139). This means that women's subjectivity is often reduced to their biological functions, which are valued in relation to their roles in reproduction. The male bias in the medical system further marginalizes women's knowledge of their own bodies, especially during pregnancy and childbirth. As Young (1984) explains, the dominant health model assumes that a healthy body is stable and unchanging—one that applies to adult men but not to women, children, the elderly, or disabled people, who are seen as “Other.”

Pregnancy and childbirth, as two distinctive bodily functions for female-coded bodies, are regarded as medical conditions for which only trained professionals are allowed the knowledge of them. Bodies considered “professional” not only have the capability to impose such knowledge but also produce it. Conversely, bodies whose subjectivity encompasses the lived experience of pregnancy and childbirth often find their knowledge undervalued. These social constructions around asserting, possessing, and generating knowledge about childbirth impact the intersubjective personhood of women by potentially marginalizing their lived experiences within the broader societal and medical frameworks, creating a sense of alienation (Young, 1984). This disconnection leads easily to violence, often unrecognized, which can subtly reorder bodily relations, alienating the birthing subject from their own personhood, power, and the communal space of childbirth.

Childbirth is an event where women can experience a state of embodied subjectivity, a situation where they can feel a connection and identification with their bodies. During pregnancy, the woman's awareness of her body becomes more complex and multifaceted, experiences herself as an active participant in a creative process. Young (1984) suggests that the pregnant woman is a dialectic subject who is intimately connected to the process of change and transformation that is occurring within her. But for such an experience to happen, one needs “free others who can give meaning to my projects” (Cohen Shabot, 2021, p. 7), which notion aligns with Beauvoir's concept of ambiguous subjectivity that combines transcendence with immanence. When giving birth, the subjects go beyond themselves (transcendence) and simultaneously are inherent within themselves (immanence).

Cohen Shabot (2021) argues that a violent birth is a solitary birth. It is a birth in which someone becomes something, where one's embodied, immanent experience does not align with the intersubjective transcendence of the event. Since the whole event is not transubstantiated as violent by others, women are condemned to navigate through this, physically and emotionally, alone. As many women in our survey reported, the experience of obstetric violence wounded deeply their relationship with their bodies (they didn’t want to touch themselves), their husbands (some led to a divorce), their newborn (difficulty in creating a mothering subjectivity), and the biomedical institution (many said that they cannot trust a doctor anymore). As we shall see later on, many women were able to digest the difficult feelings through a connection with others, either their significant ones or by advocating for their rights as birthing subjects, transcending their traumatic experience into a social movement.

## Methodology

### Participants

The study included 63 valid participants who had experienced obstetric violence in Greece. Initially, 71 responses were collected through an online survey, but eight of them were excluded since they answered negatively to the first question: “have you ever experienced obstetric violence?.” Demographic characteristics were not collected in the survey. This decision served a dual purpose: on one hand, it was an effort to ensure the authenticity of the described experiences, eliminating the self-censorship bias deriving from one's social identity awareness. On the other hand, demographics were not collected in order to ensure complete anonymity of the participants.

### Procedure

For this study, an online survey was distributed in six online, private communities. Access to these communities was provided by the administrators after anchoring the purposes of the research. Two of these online communities were about communal support and self-education on obstetric violence, while the others were parenting and children-related groups. The survey remained open for 6 days, allowing participants to respond at their own pace.

### Data Collection

The survey involved seven open-ended questions, which participants could freely choose which to answer and how long their answers would be (Braun et al., 2021). The questions were the following:

1. Have you ever experienced obstetric violence? If yes, can you describe what happened?
2. Did you receive adequate information and support before and during your childbirth experience? If not, how did this affect your experience?
3. How has the experience of obstetric violence affected your relationship with your body and your sense of bodily autonomy?
4. In what ways has your experience of obstetric violence impacted your sense of trust in your body's ability to give birth and/or care for your child?
5. How have you coped with any negative feelings/emotions related to your experience of obstetric violence and its impact on your relationship with your body?
6. In what ways has your experience of obstetric violence impacted your relationship with your partner or loved ones?
7. Are there any additional comments or insights you would like to share about your experience of obstetric violence and its impact on your relationship with your body?

The language of the survey and the data was in Greek, and any translation into English was done by the study's first author. At the end of the survey, a short paragraph with contact information of organizations in Greece which specialize in perinatal mental health and gendered violence cases was provided.

### Data Analysis and Trustworthiness

The chosen method for the analysis of the data was thematic analysis. The method can be used to analyze a wide range of qualitative data, one of it being open-ended survey responses. In line with Braun and Clarke (2006), we believe that the method invites theoretical flexibility, including phenomenology. To enhance trustworthiness, the data were carefully translated from Greek to English by the study's first author, with the second author only accessing the translated material.

#### Ethics

The data was collected with total anonymity, in accordance with law and guidelines regarding ethics in research, as research on obstetric violence may result in sensitive data. Since the research has been handled within the framework of Umeå University in Sweden, the researchers have considered regulation regarding ethical review on people (SFS nr 2008: 192 and SFS nr 2003: 460) and acted accordingly. Participants who have answered the questionnaire have been provided accurate information about the project and concerning their participation through written information (according to 13 §). There were no answers which included details which could identify anyone, the narrator's integrity was specifically considered and discussed in accordance with the General Data Protection Regulation. A secure anonymized survey service via universities in Sweden was offered to access fully anonymized material. The data have been translated from Greek to English by the study's first author, and the second author has only accessed the translated data.

## Results and Discussion

With the use of thematic analysis, combined with the theoretical framework, the following four key themes were generated: (a) practices of obstetric violence, (b) reshaping interpersonal dynamics in space, (c) disempowerment and objectification, and (d) me, we, them: dealing with injustice and doing affective work. The first theme serves as a foundational exploration, mapping out the experiences and laying the groundwork for investigating the phenomenon in Greece. The second theme delves into the organization of physical and social spaces within obstetric settings, shedding light on how these structures can limit the birthing subject's agency and control. The third theme stays with feminist arguments surrounding the objectification of the birthing woman. Lastly, the fourth theme discusses the possible psychological, emotional, and physical impact of obstetric violence on women's lives.

The results and their discussion have been integrated into the same chapter for a more fluid analysis of the data.

### Practices of Obstetric Violence

#### Unjustified and unnecessary medical interventions

The way a problem is named and constructed is essential as it gives those affected a framework to understand and acknowledge it (Chadwick, 2021). Many women in the survey talked about a plethora of interventions as being unjustifiable and unnecessary. The most common malpractice was the hastened beginning of labor with various technomedical interventions, such as artificial induction of labor, artificial breaking of the waters, oxytocin administration, artificial pains for delivery, and a more general psychological pressure to start labor although the body was not ready. In some cases, this pressure to give birth at a specific time determined by the doctor was linked to the gynecologist's scheduling convenience.

While he made a specific appointment for me, my water broke 2 days earlier, at a time that was not convenient for him as it turned out [to be] and he told me [that] to my face. … he “scolded” me for going earlier, he told me that he was going to put me in for a cesarean section. After a lot of pressure I [just let him] … I was led to an UNJUSTIFIABLE^1^ cesarean and I felt raped. (p50)

#### Traumatic and nonconsensual hospital procedures

After being admitted to the hospital, various interventions were referred to as traumatic due to their frequency, their imposed character and causing unnecessary pain. These interventions were the following: having an enema, shaving, continuous vaginal examinations, and forcible opening of the cervix (the so called “helicopter,” by the movement of the doctor's fingers), being “tied” to a bed with intravenous saline and an echocardiogram with simultaneous prohibition of consumption of food and liquids, prohibition of movement and/or being escorted by the significant other, uncontrolled entry of the medical personnel into the labor room, and refusal of providing anesthesia and/or pain relief medicine. The majority of women were not informed about what was going to happen, while none of them gave their consent for these practices.

I wasn’t asked about many things that were decided during my labor (shaving, enema, water breaking, finger check). I felt that I was very restrained in the position I would like to have while giving birth. I forced myself a lot [to comply], I was asking him if I could be in a squat position and they were asking me why and if I had done yoga [in a way that they were making fun of her]. (p69)

Interventions, such as shaving, enema, water breaking, and finger check, have been described by Davis-Floyd (1987) as the first ritual being performed once a laboring woman is admitted to the hospital, like the first step in an assembly line. It is a ritual in which the woman is “prepped.” She argues that these practices serve as a symbolic message portraying the birth as a technological process that needs to “meet production and quality control demands” (Davis-Floyd, 1987, p. 482). The laboring woman and her body are condemned into a Cartesian self, separating the mind from the body. The birthing body is no longer part of the woman's subjectivity, but is now a distinct process relegated to the “professional bodies” job experience. Like the products in a production line, birthing bodies need to comply with the hands of the workers (Davis-Floyd, 1987; Young, 1984). Although some of these practices may not be considered traumatic on their own (like shaving or the vaginal examination), it is the way they are practiced and imposed on birthing subjects that makes them part of the obstetric violence concept.

#### Violent and outdated medical practices

Other interventions, that were highlighted in many narratives and are far more problematic and even prohibited in nowadays medical practice, are the unnecessary episiotomy/perineotomy, the use of forceps and/or ventouse (vacuum delivery), the Kristeller maneuver and the husband's stitch. All these techniques have been proven to be dangerous both for the mother and the baby, while some can cause significant damage to the female body affecting many of its functions such as urination, defecation, and sexual pleasure, leading to various infections postlabor and further psychological damage (Diniz & Chacham, 2004; Eason & Feldman, 2000; Malvasi et al., 2019). As one of the women says:

A Kristeller [maneuver], the use of a ventouse, a large perineotomy and the husband's stitch were performed (they hurt for about two years during sexual intercourse). Of course, all these happened without information or consent, without an epidural. Just thinking about them, I get a knot in my stomach even though it's been 5 years [since then]. (p17)

#### Psychological abuse and disruption of maternal bonding

Behaviors such as causing frustration and fear with misleading and unsupportive comments, being verbally abusive (ironic, derogatory, insulting, and disheartening comments) and a general prohibition of expressing and relieving the pain through shouting, moaning, and crying, were repeatedly reported. Finally, practices that made the bond between the mother and the infant difficult were shared, such as the instant mother–infant separation, the instant ligation of the umbilical cord, not supporting women during breastfeeding, not informing mothers of the infants' health condition and prohibiting them from seeing their babies. Several stories point to how these practices were accompanied by disrespectful behavior by the medical personnel:

They took it [the baby] from me aggressively without really explaining why. They wouldn’t let me see it and they barely took the breast milk [in order to feed the baby]. (p37)

### Reshaping Interpersonal Dynamics in Space

Merleau-Ponty (1962) pointed out that our relationship with the world is formed through a reciprocal and integrating experience of the mind and body as a whole. It is our situated, embodied experience as human subjects that allows us to be part of the spatial world, creating a bidirectional relation between ourselves and the physical spaces. Thus, the physical spaces acquire another dimension, the sociocultural one. Spatiality refers to the ways in which physical space, environment, and location shape social relations and experiences. In the context of obstetric violence, spatiality can be about both the hospitals/maternity units as physical spaces (e.g., the medical equipment, the room's layout, etc.) and the ways people form and organize their social interactions in them.

I was afraid of the unknown and they took advantage of it, creating more fear so that I would need them and they did whatever they wanted since I was now their hostage. (p9)

I was taken upstairs, restrained on the stretcher, lying down, and they wouldn’t let me move. The doctor would come, check my dilation, and leave. (p8)

During the surgery, I felt like I was in a cafeteria. And when they were done, I was left in the room for over an hour because there was no order to transfer me. I was cold and far from my baby, who needed me so much. (p50)

The above voices are just a minor selection of all the narratives. Many of the experiences described in these quotes are repeatedly echoed in the stories of other participants. These quotes describe the physical and spatial interactions and restrictions imposed on birthing women: being confined to beds, restrained, deprived of movement, and left alone in cold rooms surrounded by machines. The comparison of the operating room to a cafeteria by participant 50 is particularly striking since, instead of being a space of care and attentiveness, the surgical room becomes a place of detachment and disempowerment. This situation further reinforces the feeling of depersonalization, where the person in labor is seen as just another case that has to be handled. A body connected to machines:

Due to an incorrect cardiotocography reading, they rushed me into the delivery room, claiming that the baby was having arrhythmias. And I say incorrect because, in hindsight, I realized that the machine wasn’t making proper contact. The midwife got up, left, and abandoned me alone with the machine. (p37)

This experience exemplifies how the reliance on a technological machine became the determining factor in the course of labor, even when it was providing inaccurate readings. This participant (p37) further described how the medical personnel insisted on using this malfunctioning device and demanded no movement of her in order to get accurate results of the cardiotocography. The reliance of the medical staff on a device reinforces the idea that birth is a process managed by technology. It is a striking example of how medicalized birth spaces operate under rigid power dynamics that position women as passive subjects within a technological process. The birthing body is conceptualized as a body that needs repair, a body that enters a production-like technological process and is stripped of its agency and autonomy (Davis-Floyd, 1987). Based on this social construction, the relations between the expectant mother and the medical personnel are built upon and organized. The birth is now guided entirely by the medical personnel. This is a power dynamics repositioning that participants in the survey often expected to happen. In their narrations, they used adverbs such as “of course,” “obviously,” “absolutely,” “definitely,” “sure thing” while describing what happened.

Obviously the doctor cut me. (p35)

Of course, you feel that you have no control over any decision regarding the birth of your own child. (p58)

A recurring narrative was the organization of the physical space and the social interaction in such a way that caused women psychological pressure to be compliant and docile with the medical guidelines. Through the enforcement of various medical practices, an emotional “war,” the dire conditions of the room and the exams, and the narrative of the medical personnel as the only ones who *can* know, many women admitted that they were led to interventions and decisions, such as a cesarean section, without actually wanting them. A feeling of indignation at all those receiving maltreatment was also reported, as illustrated in the following story:

The nurses were speaking badly. And my gynecologist was always very nervous and [he] would shout, making me afraid to ask something or repeat a question because he would get angry … Various gynecologists and trainees would enter the room and put their hands on me three times an hour to check if there was adequate dilation, causing me to cry and feel dirty. Traumatic experience! In the end, I had a cesarean section and said thank God I will not experience this humiliation again, as I will have cesareans from now on. (p40)

### Disempowerment and Objectification

Testimonies of a lack of room for manoeuver and autonomy can be found in all responses to the survey. Although lack of information was not always reported (some women mentioned that they were adequately informed about what was going to happen), a feeling of helplessness, being “absent” and not being heard was constantly there.

An interesting recurring pattern in participants’ use of language was observed: they consistently positioned themselves as passive recipients of actions carried out by others. For instance, expressions like “they sewed me up,” “they ripped me off,” “he puts his hand on me,” “they didn’t let me,” “he pushes me to break my water,” etc., suggest a strong narrative of power imbalance in the healthcare provider–woman relationship, where the healthcare provider has the power to make decisions without the woman's input. This lack of agency can be a form of violence that perpetuates a disconnection between women and their bodies, leading to feelings of disempowerment and trauma. As the participant describes below, an incompatibility of the lived experience and the intersubjective transcendence of the event leads to an isolated birth; a birth where physical and emotional repercussions are navigated alone (Cohen Shabot, 2021). The quote below highlights this isolation. Although several women may eventually move past the experience, many mention how the memory remains indelibly etched within them.

I would say that although it is known that women understand the bad behavior during childbirth, they do not have the autonomy and control over the situation to react. This is something that a woman can bypass later, but she will not forget. (p55)

During childbirth, several participants experienced a natural urge to connect with their bodies, which created an inner conflict with the external treatment they received. The stories below testify to degrees of violence normalized in the medical personnel's contact with the laboring mother, and illustrate everything from mocking the desire for a birth plan to destroying one's body without commenting on what happened. But it is also about the relationship of the person giving birth to the child from their body, and the patriarchal disregard of this relationship as one more practice that reinforces the birthing body as other.

When I went [to the doctor] with a birth plan, the doctor laughed. (p5)

I said that I don’t want an episiotomy and the doctor just laughed and it did it anyway. (p63)

When I took the baby from the unit I tried to breastfeed and the midwife told me laughing that this baby won’t breastfeed. It wasn’t so much the irony in her tone as the contempt for my body. I was so sure I would make it and she collapsed everything. She made me feel small and incapable. (p54)

Rich (1995) talked about the early relationship between the mother and the infant, highlighting the idea of them as a unified entity even after birth. This idea results in an interdependent care provision, with which this bond is acknowledged and respected. During a technomedical obstetric routine, the mother–infant bond is neglected as if the baby does not belong to its mother anymore but rather to the institution in charge (Chadwick, 2021; Davis-Floyd, 1987). This immediate “scheduled” separation of the baby is characterized as traumatic by a lot of women in the survey and is considered part of the obstetric violence concept.

[I said that] I want to delay the clamping of the umbilical cord and [the doctor] pretended not to hear me. (p63)

In the hospitalized setting, the infant and the mother are not only considered two different entities with different needs, but their bond and the mother's efforts to bond are ridiculed by the medical personnel. This is especially pronounced during breastfeeding. Like in the quote of Participant 54 above, the midwives neglected to offer any assistance to the mother and instead assumed an authoritative stance, asserting their knowledge and expertise regarding the procedure and how it should be carried out.

Young (1984) described the expectant mother as an active participant in a creative process, a dialectic subject that can be both the source and subject of change. While Young's framework brings a positive light to women's agency and innersubjective transformative potential, obstetric violence strips women of their subjectivity by constantly and forcefully objectifying them, leading to a sense of alienation. Women are disempowered and marginalized within the childbirth process.

Cohen Shabot (2016), following Rich (1995), associated this feeling of alienation with the feeling of being raped. She mentions that a total deprivation of subjectivity, a total violation of bodily autonomy, a total loss of control and agency, and a sense of self-blame are often discussed in cases of obstetric violence and rape. One of her main arguments is that rape and the violence it entails are the most potent tools of patriarchy to subjugate female bodies and reinforce their position as objects. The violence that perpetuates both rape and obstetric violence serves as the way in which patriarchal societal “tames” “a loud and subversive embodied subjectivity” (Cohen Shabot, 2016, p. 234). Short lines, written by two birthing subjects (echoing many others) illustrate this:

I felt literally violated in every cell of my body. (p39)

I felt as if I had been raped without knowing what had happened to me. (p23)

### Me, We, Them: Dealing With Injustice and Doing Affective Work

I would like a way to be found for all obstetrician-gynecologists who have used violence to be exposed, and perhaps there should be sanctions. There are many, many of us who have suffered such violence and worse. (p39)

Many feelings have been described in the survey, both distressful and empowering. As the participant above protests, several others have articulated an urging desire for social justice in order to lead to an inner catharsis. Since childbirth can have a communal character, obstetric violence not only severs the connections birthing subjects have with their bodies, but damages the connections with the world around them as well (Cohen Shabot,^2021^). These connections are essential since people are situated, embodied entities experiencing life through and with others (Merleau-Ponty, 1962). As violence can harm interpersonal connections, community can play a crucial role in the healing process. A connection with the newborn—primarily through breastfeeding—and the other parent, though challenging at times, acted as an empowering factor in women's lives.

In this theme, we’ll explore the interplay of distress and empowerment, the ways of dealing with negative emotions and the relationship with the significant other, ultimately concluding our discussion of the experience of obstetric violence and its aftermath.

#### Distress versus empowerment

Several women reported experiencing a range of negative emotions following experiences of obstetric violence. They referred to feelings of sadness, frustration, and pain, as well as sexual dysfunction and/or lack of desire for sexual contact. Many women talked about feeling deceived by their doctors and experiencing a lack of trust in medical professionals. An insecurity and difficulty in trusting their own experiences and bodies was reported, followed by a general feeling as if they had lost their dignity and femininity, and that their autonomy and self-esteem had been taken away from them. This experience is vividly portrayed in the following participant's answer, where she draws a parallel between herself and a wild animal:

At first, I felt empty … Without any emotion, not even for my own child … They attacked me even more after birth for my decision to exclusively breastfeed, so I felt like a wild animal … I felt like I lost my dignity, that I was raped, that I was ridiculed, and that they even stole my femininity since I couldn’t even enjoy sex. (p17)

The experience of obstetric violence resulted in both physical and psychological trauma, as mentioned by the participants, a finding that agrees with the bibliography (e.g., Çetin et al., 2023; De Souza et al., 2017; Martinez-Vázquez et al., 2022). Some women confided that they were diagnosed with postpartum depression, while a range of emotions—such as anxiety, anger, fear, denial of future childbirth, overeating, discomfort with their own bodies, feeling helpless and unsupported, as well as detached from their own bodies and their babies—were present. The physical consequences of obstetric violence (for instance, pelvic floor damage and C-section scars) further contributed to negative feelings of distrust and alienation over their own bodies.

During the first 20 days, maybe even a month, I felt disabled, as if my legs had been cut off. It took me maybe more than two months to be able to touch the incision point [from C-section] without bursting into tears and more than five months to stop crying altogether when I washed or dried the area. However this feeling of disability often returns, even almost eight months later, I don’t feel it as a part of me, I don’t have any sensation when I touch it. (p32)

It [obstetric violence] caused me postpartum depression and then I was left with a lot of anger and pain. (p2)

Some wondered how their experience would be if they had the chance to have a vaginal birth without violence and if their bodies would be capable of bearing it. Rich (1995) acknowledged this mythical veil around childbirth, that women view labor as a mysterious and quite painful bodily function, in which the female body experiences the peak of its capabilities. The birthing woman not only anticipates experiencing extreme pain, but the unknown nature of the process causes feelings of fear, anxiety, and frustration. Will the body be proven capable of overcoming this trial?

I am curious about how my childbirth would have been if I was ultimately given the opportunity to have a vaginal delivery, if I would endure the pain and to what extent it would have been a more traumatic experience than what I have now. (p5)

Interestingly, a significant number of participants expressed a feeling of empowerment and psychological resilience in the aftermath of these experiences. They talked about their need to respect their bodies and know their limits. They felt that their bodies were not to blame for what happened, and they tried to be aware and vindicate their needs and desires in future childbirths. It is intriguing how a sense of owning one's body comes after its agency is taken away, especially regarding women's bodies; how women need to fight for their right to their bodies, like it is the only way patriarchy allows them to become subjects. As one of them wrote:

I respect my body more now. I didn’t know before that I had a ‘right’ to my body and my childbirth. (p42)

#### Dealing with negative emotions

Dealing with frustration and sadness after experiencing obstetric violence can be a challenging experience. What seemed to be quite helpful for the majority of women in the survey was to actively seek healing. Various ways to cope with traumatic feelings were described, such as a turn to the community through parenthood groups and breastfeeding clubs, connecting with friends and family, visiting church and praying, being part of online communities, etc. All these approaches can provide a sense of belonging and understanding. After all, feminist theorists suggest that obstetric violence is not just an individual issue but a systemic one that is deeply rooted in society's patriarchal and sexist structures (e.g., Chadwick, 2021; Cohen Shabot, 2021; Van Der Waal et al., 2022). Therefore, addressing any form of gendered violence requires not only individual healing but also collective action and communal warmth and care. Many women felt the need to self-educate and act as ambassadors for other women in order to prevent future instances of obstetric violence. Finally, embracing the identity of motherhood and connecting with the baby (especially through breastfeeding) was portrayed as a painkiller (Oliveira & Penna, 2017). To jointly put words on the experience, finding communities, and reconnecting with their bodies through their child were described as invaluable:

I discuss them [the feelings] with my partner who supports me a lot. The Facebook groups where mothers share similar experiences have helped me a lot. (p4)

I have also taken antidepressants in combination with [psychological] monitoring by a specialist, but more than anything I can say that my spiritual leader and the Divine Communion helped me. (p37)

Extended breastfeeding of my baby helped a lot in regaining the connection between it and me. To fill the gap left by its abnormal birth. (p64)

#### The relationship with the partner

In this subtheme, we identified two opposite experiences regarding the relationship of the woman with her significant other. Most women reported having negative consequences in their intimate relationships, since they felt that others couldn’t fully understand what they had gone through. Moreover, the husband's negative comments about their bodies, his absence during childbirth and his lack of empathy led to emotional distancing. For some participants, obstetric violence led to a breakdown in the relationship, with some experiencing fear that their partner would no longer want to touch them or that there would be pain during sexual intercourse. These testimonies highlight the long-lasting effects of obstetric violence, revealing it to be something more than a singular act of violence that starts and ends in the hospital.

Among other things, this whole experience distanced me from my then-husband, who couldn’t and maybe didn’t want to understand what I went through. He questioned the extent of my trauma. Eventually, we got divorced. (p50)

On the other hand, a handful of women reported that the healing process brought them closer to their partner as they shared the trauma together and found comfort in each other. Some found that their partner supported them in their desires for future childbirth, such as having a home birth.

My partner was fully supportive of the home birth, he was there constantly, his presence helped me a lot, his love, care, and the fact that he did not hesitate at any moment throughout the process. I remember his behavior and I am moved. (p20)

What is crucial to emphasize here is that obstetric violence isn’t isolated to a single moment or individual. It is a whole experience that affects the woman, the partner, the relationship between them and the relationship with the newborn. Given the extensive reach of this violence in both time and space, it demands urgent attention and recognition.

## Conclusion

Obstetric violence is a pervasive global issue that can occur at any stage of pregnancy, childbirth, or postnatal care (Castro & Frías, 2020; WHO, 2014). The narratives shared by the participants in this study highlight the entrenched power imbalances within the Greek healthcare system, which seem to have been exacerbated by the historical transition from home births to hospital-based deliveries (Lefkarites, 1992). The women's accounts in this study illustrate a wide range of violations, underscoring a profound asymmetry in the relationship between healthcare providers and birthing individuals. This power imbalance not only denies women agency over their own bodies and experiences but also perpetuates a patriarchal framework in which medical authority is privileged over lived knowledge (Cohen Shabot, 2021). By emphasizing the dynamic nature of subjectivity, obstetric violence can be understood as deeply embedded in embodied experiences and relational dynamics. Furthermore, it is shaped by dominant societal narratives that dictate the assertion, possession, and dissemination of knowledge about childbirth (Young, 1984).

While this study offers valuable insights into the experience of obstetric violence, it is not without limitations. Although the number of participants is already enough for a qualitative study, it may not fully represent the broader diversity of women's experiences. The chosen data collection method (a qualitative online survey) may provide flexibility and ensure total anonymity, but it may be restrictive for more in-depth responses and the nonverbal aspects of communication that can be captured in an interview setting. Future research could explore the impact of obstetric violence on the shaping of a gendered parental role and power dynamics within families. Additionally, it would be valuable to investigate how such violence affects women's decisions to have more children and the alternatives they consider for subsequent births. Another aspect that could be researched is the perspectives of medical staff in order to better understand the institutional dynamics and sociocultural norms during their training and professional practices. Finally, the silence of the supporting medical personnel (e.g., midwives, nurses, etc.) is one more important issue to explore, as it may reveal the ways in which institutional hierarchies pressure the concealment of such phenomena.

If we look closely at qualitative research on obstetric violence, which has been done in different parts of the world, we find that the narratives of the women share many similarities. This means that, besides the cultural differences of each region, this form of violence seems to follow common patterns of power dynamics, disempowerment, and resistance. For instance, similar findings are echoed in Turkey, where Avcı and Kaydırak (2023) identify structural neglect and emotional harm as central features of obstetric violence, derived from a phenomenological analysis of women's lived experiences. Sharon and Alnabilsy (2024), focusing on Arab and Jewish women in Israel, add further nuance by analyzing the long-term psychological consequences of obstetric violence and women's coping strategies, while also foregrounding how structural and ethnic inequalities mediate the experience and aftermath of such violence. Besides the religious differences, participants reported similar consequences on a physical and an emotional level (e.g., pain, injuries, inflammations, sadness, oversensitivity, emotional detachment, etc.). Moreover, they talked about the negative consequences to the spousal relationship and to the healthcare system and healthcare professionals (Sharon & Alnabilsy, 2024).

What can be learned from the experiences of Greek women may not be entirely different from what can be learned from women in other countries; however, the importance of the current study lies in recognizing the shared patterns of the prevalence of malpractices, the structure of power hierarchies within the maternity wards, and the ways in which knowledge is produced and maintained. Together with our study, previous research underscores that obstetric violence is not merely the result of individual malpractice, but deeply embedded in institutional cultures and broader sociopolitical conditions, often intersecting with gender, ethnicity, and class (cf. Davis, 2019). Since research on obstetric violence is a relatively new study area, the current endeavor is unique in its challenge to the production of biomedical knowledge by amplifying women's embodied experiences and giving voice to those often silenced.

Finally, we firmly believe that, despite the challenges discussed in this study, a hospitalized birth can be a positive and empowering experience for many women, even in the face of obstetric violence. A variety of things can contribute to such a positive experience, such as prenatal classes and self-education, access to pain management options, having a supportive birth partner, a calm and supportive birth environment, etc. But the most important factor is following an approach and care that is centered on the birthing subject, meaning the prioritization of a woman's needs, wishes, and desires; being respectful of her autonomy and providing the space to feel in control of her body and her experience (Bradfield et al., 2019; Hodnett, 2002).

Our study shows a willingness to speak out about obstetric violence—sometimes being a killjoy (cf. Ahmed, 2010)—to refuse to be silent and also to contribute to fewer people bearing witness to similar experiences. Several voices in our study are in line with an emerging mobilization to recognize and advocate for significantly improved birth experiences and good obstetric care. Recently, a new movement in obstetrics has developed, the humanized childbirth movement, which is particularly evident in South America (Marques et al., 2020; Rodrigues et al., 2021). According to this movement, knowledge around the nonpathological character of childbirth is disseminated during professionals’ training, focusing on the value of a natural birth and the shift in obstetrics ethics toward more emotionally engaged and respectful care. Better health practices are reinforced, centering on creating a strong bond between the birthing subject and doctors, through a sympathetic and affective dialog (Rodrigues et al., 2021). Moreover, this movement calls for obstetric care that aligns with recent research and evidence-based methodologies, aiming to minimize (primarily) women's suffering, eliminate unnecessary and inappropriate practices, and prevent the misuse of healthcare resources (Marques et al., 2020).

## Notes

1. The formatting of participants' answers remained intact. The only alteration regarded the translation from Greek into English.
